# The impact of pregnancy loss on men’s health and wellbeing: a systematic review

**DOI:** 10.1186/s12884-017-1560-9

**Published:** 2017-11-15

**Authors:** Clemence Due, Stephanie Chiarolli, Damien W. Riggs

**Affiliations:** 1 School of Psychology, The University of Adelaide, Adelaide, South Australia 5005 Australia; 2 School of Social and Policy Studies, Flinders University, GPO Box 2100, Adelaide, South Australia 5001 Australia

**Keywords:** Men, Pregnancy loss, Miscarriage, Stillbirth, Ectopic pregnancy, Systematic review

## Abstract

**Background:**

Research indicates that men’s psychological and physical health outcomes after pregnancy loss differ from those of women. Our goal was to identify all literature with a focus on men’s experiences of pregnancy loss in order to outline current evidence concerning men’s wellbeing.

**Methods:**

A systematic review of literature on men and pregnancy loss was undertaken following the Preferred Reporting Items for Systematic Reviews and Meta-Analyses (PRISMA), Joanna Briggs Institute (JBI) and Social Care Institute for Excellence (SCIE) guidelines. Literature was sourced from PsycINFO, PubMed, Scopus, CINAHL, and Google Scholar. Inclusion criteria were 1) studies that focused on pregnancy loss (including miscarriage, stillbirth, and ectopic pregnancy, 2) that men’s voices were specifically represented, and 3) that studies were of primary data.

**Results:**

A final sample of 29 articles was identified, of which 16 were quantitative, 10 qualitative, and 3 mixed methods. Quantitative and mixed methods studies indicated that while men tended to have less intense and less enduring levels of negative psychological outcomes than women, they are more likely to engage in compensatory behaviours, such as increased alcohol consumption. Qualitative studies indicated that men often feel that their role is primarily as a ‘supporter’ to their female partner, and that this precludes recognition of their own loss. These studies also reported that men may feel overlooked and marginalised in comparison to their female partners, whose pain is typically more visible.

**Conclusions:**

Further research is needed on men’s experiences of pregnancy loss, focusing on cultural differences. The experience of gay and/or transgender men who face pregnancy loss is overlooked in the literature to date.

## Background

Pregnancy loss affects many people every year, with miscarriage occurring in approximately 15–50% of all pregnancies [1], the majority of which occur before a pregnancy is formally recognised [2]. In addition, in 2015 an estimated 2.6 million babies were stillborn [3]. Miscarriage is defined as the unintended termination of pregnancy resulting in foetal death that occurs prior to 20 weeks of gestation, and stillbirth is the death of the foetus after 20 weeks or after reaching 400 g in weight [4]. Ectopic pregnancy is a pregnancy in which the fertilised ovum implants outside the uterine cavity [5].

There are a significant body of studies detailing health and wellbeing outcomes for heterosexual women who experience a pregnancy loss, and how such women can be best supported [6–9]. However, as pregnancy is still arguably considered primarily a ‘women’s issue’ [10], fewer studies have considered the impact of pregnancy loss on the health and wellbeing of men. Furthermore, the literature on men tends to focus almost exclusively on heterosexual cisgender men, with research on the impact of pregnancy loss on gay and/or transgender men lacking. This lack of research is particularly problematic since available research details a range of potential health impacts for men following pregnancy loss, including disenfranchised grief, anxiety, depression, and a tendency to resort to avoidance behaviours such as alcohol and drug use [1, 7]. Taken together, men’s experience of pregnancy loss is a key area of concerning for healthcare professionals working with men and their families.

The present paper provides a systematic review of research findings from all empirical research designs with regard to men and pregnancy loss. It aims to describe the nature and characteristics of current research into the impact of pregnancy loss on men, and in so doing provides a solid base for future empirical research that addresses gaps in the literature.

## Method

Preferred Reporting Items for Systematic Reviews and Meta-Analyses (PRISMA) [11] and Joanna Briggs Institute (JBI) methods [12] were drawn upon in the collection and analysis of articles, as well as qualitative data synthesis and analysis processes from the Social Care Institute for Excellence (SCIE) Systematic Research Review Guidelines [13].

### Inclusion criteria

Any peer-reviewed study published in English between 1995 and 2016 was included for review in order to capture all recent research. Inclusion criteria were that studies 1) aimed to investigate the impact of pregnancy loss on men’s health and wellbeing with pregnancy loss referring to miscarriage, stillbirth and ectopic pregnancy (studies which focused on perinatal loss in the early neonatal period were excluded), 2) included either a focus on men or included men’s voices separately (i.e., in studies that focused on both men and women), 3) were empirical, involving primary data collection. The search also identified a number of studies that focused on the impact of pregnancy loss on men’s health and wellbeing in the context of a subsequent pregnancy, and these papers were assessed as meeting the inclusion criteria and were therefore included in the review. Studies which investigated the impact of both elective termination and termination due to foetal abnormality were excluded as previous research suggests that these may be experienced differently to an unplanned pregnancy loss [14, 15].

### Search strategy and data extraction

An initial search of PsycINFO was undertaken to identify subject headings and potential keywords. The subject headings and potential keywords identified in this initial search were: spontaneous abortion, pregnancy loss, miscarriage, perinatal loss and stillbirth along with human males, husbands, spouses, men, males, fathers and paternal. A second search was then undertaken in PsycINFO using these keywords, subject headings and index terms. The search was then undertaken in PubMed, Scopus, CINAHL, and Google Scholar, and reference lists selected for full text review were searched for articles of relevance. Example search strategies from PsycINFO and PubMed are outlined in Tables 1 and 2. All studies identified could be accessed by the authors without contact with individual researchers. The final date for inclusion was end of July 2016.

**Table 1:** PsycINFO search strategy

Pregnancy loss/miscarriage	Men’s health outcomes
Spontaneous abortion.sh OR spontaneous abortion*.mp OR Pregnancy loss*.mp OR miscarriage*.mp OR Perinatal loss*.mp OR Stillbirth*.mp	human males.sh OR husbands.sh OR spouses.sh OR husband*.mp OR Men.mp OR Male.mp OR Males.mp OR father*.mp OR paternal.mp

The * acts as a truncation symbol, whereby the search includes all possible endings for the term

**Table 2:** PubMed search strategy

Pregnancy loss/miscarriage	Men’s health outcomes
Abortion, habitual [mh] OR Abortion, spontaneous [mh] OR Stillbirth/psychology [mh] OR Miscarriage/psychology [mh] OR miscarriage*[tiab] OR Perinatal loss*[tiab] OR Stillbirth*[tiab]	Men’s health [mh] OR Fathers/psychology [mh] OR Spouse*[tiab] OR husband*[tiab] OR Men [tiab] OR Male [tiab] OR Males [tiab] OR Father*[tiab] OR Paternal [tiab]

The * acts as a truncation symbol, whereby the search includes all possible endings for the term

The initial search of PsycINFO, PubMed, CINAHL and Scopus generated 3976 results. After reviewing all abstracts, 108 articles were retrieved by the first author, and the full texts of these articles were reviewed by all authors. The primary reason for exclusion of the remaining 3868 articles was a focus on women’s medical and gynaecological issues of pregnancy loss, rather than men’s health outcomes. Studies were also excluded if they reported on women’s perceptions of the impact of pregnancy loss on men, rather than specifically including men as study participants. Of the 108 articles retrieved for full-text examination, 27 met all inclusion criteria and were agreed upon by all three authors. A further two articles were retrieved from Google Scholar after reviewing reference lists, both of which met the inclusion criteria, giving a total sample of 29 articles. Three of these studies were mixed methods in design, and for synthesis purposes, these were incorporated into the reporting of the quantitative studies as the study designs were primarily quantitative. The details of all 29 included studies are outlined in Appendix.

Overall quality and risk of bias was assessed using the Cochrane Collaboration’s tool for assessing bias. The second and third authors reviewed the studies and reached consensus, however no study was excluded on the basis of quality given the low number of studies that met the inclusion criteria. The following section contains discussion of overall risk of bias in this body of research. Finally, a formal meta-analysis of the quantitative studies was not conducted due to the heterogeneity of studies in terms of design, study populations and outcome measures.

The first author extracted important characteristics of the studies using a predesigned table. This information included: country where the research was conducted, date of publication, study design, number and characteristics of participants, and psychological and physical health outcome measures. This information was cross-checked by the second author.

## Results

As noted above, 29 studies were included in this review from 108 reviewed full-texts and 3976 search results. Sixteen of the included articles were quantitative, 10 qualitative, and three used a mixed methods design. Two of the studies utilised the same data and population but reported on different aspects of the findings, so they were both included. Fifteen studies examined the impact of a pregnancy loss on both men and women, and aimed to identify any differences and similarities in their experiences. Nine of the studies solely attempted to identify the impact on men, and a further four studies aimed to explore the experience of a subsequent pregnancy for men after experiencing a prior pregnancy loss, with two of those studies investigating both men and women.

### Description of quantitative and mixed methods studies

Table 3 provides details of the key characteristics of the 19 quantitative and mixed methods studies. All of the male participants were heterosexual and cisgender. Of the 16 solely quantitative studies, 12 aimed to compare heterosexual couples’ psychological experiences of a pregnancy loss, while four aimed to measure only men’s psychological reactions. Four of these studies involved men whose partners were currently pregnant following a prior loss.

**Table 3:** Characteristics of the quantitative and mixed methods studies

	Number of studies^a^
Year of publication	
1995–2000	11
2001–2005	3
2006–2010	4
2011–2016	1
Sample size of males included^b^	
< 30	5
30–60	6
60–100	2
101–300	4
301–400	2
Region of study	
Australia	3
Europe/United Kingdom	6
Canada/United States	6
Asia	2
Recruitment	
Hospitals/clinics	11
Private Practices/General Practitioners	2
Education classes	1
Pregnancy loss support group	1
Pregnancy loss clinic	2
Other	4
Time since pregnancy loss	
0–8 weeks	6
8 weeks-6 months	6
6 months-1 year	4
1 year-2 years	5
2 years +	3
Time of gestation at which loss occurred	
5–12 weeks	5
13–20 weeks	6
21 weeks–30 weeks	3
31 weeks–40 weeks	1
> 40 weeks	1

^a^ If numbers total more or less than 18 then the characteristics of a study were unknown or were relevant in more than one category

^b^ The sample size for studies assessing couples is halved so that the results for men only are displayed

The three studies that used a mixed methods design were primarily based on a self-administered quantitative questionnaire that also incorporated a small number of open-ended questions [16, 17]. One mixed methods study utilised a self-administered questionnaire which was supplemented with semi-structured interviews with 10 out of 126 male participants [18]. The aims of each of these studies were relatively different: one investigated grief responses of couples following a pregnancy loss and the adequacy of support; one aimed to examine the emotional, social and physical effects of a pregnancy loss on families; and one examined the psychological impact of a pregnancy loss on males.

Most of the quantitative or mixed methods studies were undertaken in the European Union (EU; *n =* 6) or Canada or the United States (US; *n* = 6), and participants were most commonly recruited through referrals from hospitals or clinics (*n =* 11), meaning that they were convenience samples. Sample sizes of male participants included in the studies ranged from 17 to 332 but were most commonly between 30 and 60. Time since the pregnancy loss occurred and when the men participated in the research varied, but the majority of studies (*n =* 6) interviewed participants within 8 weeks of the pregnancy loss occurring. The time of gestation at which the pregnancy loss occurred ranged from five to 44 weeks, and was between 5 and 20 weeks in the majority of studies.

Table 4 provides details of the psychological instruments used to measure outcomes in the quantitative and mixed methods studies. The psychological variables that were measured most often were depression, anxiety and grief. The most commonly used instruments were the Beck Depression Inventory (BDI) [19], the State-Trait Anxiety Inventory (STAI) [20], the Perinatal Grief Scale (PGS) [21] and the Impact of Event Scale (IES) [22].

**Table 4:** Instruments used to measure outcomes in the quantitative and mixed methods studies

	Number of studies^a^
Grief	*8*
The Grief Experience Inventory-Loss Version	1
Munich Grief Scale	1
Perinatal Grief Scale	6
Grief Experience Inventory-Perinatal	1
Depression	*10*
Center for Epidemiological Studies-Depression Scale	1
Von Zerssen Depression Scale	1
Beck Depression Inventory	6
Anxiety	*8*
Stait-Trait Anxiety Inventory	5
Hospital Anxiety and Depression Scale^b^	2
Pregnancy Outcome Questionnaire	1
Delusions Symptoms State Inventory^b^	1
Stress	*5*
PTSD-I Interview	1
Impact of Event Scale	4
Coping	*2*
Coping Response Inventory	1
The Coping Scale for Adults	1
Marital Satisfaction/Relationship	*4*
Quality Marital Index	1
Golombok Rust Inventory of Marital Satisfaction	1
Intimate Relationship Scale	1
Partnership Questionnaire	1
Dyadic Adjustment Scale	1
Other	*6*
Prenatal Attachment Inventory	1
The Complaints List	1
General Health Questionnaire	1
Ego Strength Scale	1
Life Experiences Survey	1

^a^ If numbers total more or less than 18 then the characteristics of a study were unknown or were relevant in two groups

^b^ This instrument is listed under ‘anxiety’ but is also counted under ‘depression’

### Outcomes of quantitative and mixed methods studies

All of the studies which compared men’s and women’s psychological experiences found a difference in response patterns. It was generally found that while men reported many of the same feelings of grief, depression, stress and anxiety as women, they tended to have less intense and less enduring levels of these psychological outcomes than did women [7, 23–30]. The studies which examined men whose partners were pregnant following a loss indicated similar results, in that anxiety and depressive symptoms tended to be high antenatally but these were likely to subside postnatally [31–33]. Kagami et al. [25] found that women showed significantly higher levels of depression and anxiety following a pregnancy loss compared to men, and this was significantly associated with poor quality of the marital relationship.

Similarly to previous research concerning women, however, gestational age at time of loss was not related to the health or wellbeing outcomes of men [26, 28, 29, 31, 32]. Instead, and again similarly to research with women, whether or not a pregnancy was planned or welcomed was found to be a key indicator of levels of psychological distress [26].

Some of the quantitative studies reported that men were typically hesitant to disclose their feelings, had elevated results on avoidance scales, had difficulty accessing support, and utilised varying coping strategies, including focusing on work as a distraction [23–25, 33–36]. However, and in contrast to other quantitative studies, men in Johnson and Puddifoot’s [18] mixed methods study scored high in grief similar to that expected for women (that is, the entire sample of both men and women had a high mean grief score). Also supporting this were the results of Conway and Russell’s [16] mixed methods study, where men scored significantly higher than their female partners on the three subscales and overall scores on the Perinatal Grief Scale. Similar to other studies on grief [37], however, men scored lower in ‘active’ grief than women – incorporating sadness, missing the baby and crying for the baby – further supporting the idea that men tend to suppress outward signs of grief. Also consistent with other studies were themes that emerged from the analysis of open-ended questions, including the perceptions of others minimizing the loss, active-avoidance as a coping strategy, and the meaning generated by being able to view ultrasound images which was associated with higher levels of grief.

Only two quantitative or mixed methods studies touched on non-psychological impacts of pregnancy loss. Vance et al. [38] examined patterns of alcohol use following a pregnancy loss, and found that 7–12.3% of bereaved fathers met the criterion for heavy alcohol usage compared to 4.7–5.8% of non-bereaved fathers. Turton et al. [32] assessed the psychological morbidity of fathers during a pregnancy subsequent to a stillbirth, and found that nine out of 34 fathers who were non-drinkers reported increased alcohol consumption, seven out of 38 fathers reported they had used prescribed medication to enable them to cope, and three fathers reported that they had used illegal drugs. DeFrain et al. [17] also examined the social and physical effects of pregnancy loss in their mixed methods study. They found that drug and alcohol use had increased in 7% of households (*n* = 12), and that family violence related to the loss occurred in 3% of households (*n* = 5). They also found that 6% of parents (*n* = 10) moved from their homes or communities to escape from the painful memories and/or friends or relatives who they felt were insensitive to their loss.

### Description of qualitative studies

Table 5 provides details of the key characteristics of the ten qualitative studies. In contrast to the quantitative studies, only three of the ten qualitative studies aimed to compare couples’ experiences, while the remaining seven focussed solely on men’s perspectives (two of these involved men whose partners were currently pregnant following a prior loss). Most studies were published between 2001 and 2005 (*n =* 6), which may indicate attempts to address gaps that were uncovered in earlier quantitative studies. Most of the studies were undertaken in the EU (*n =* 4) or Australia (*n =* 3), and participants were most commonly recruited through snowball sampling (*n =* 5) and through referrals from hospitals and general practitioners (*n =* 6).

**Table 5:** Characteristics of the qualitative studies

	Number of studies^a^
Year of publication	
1995–2000	1
2001–2005	6
2006–2010	2
2011–2016	1
Sample size of males included^b^	
1–5	3
6–9	3
10–14	4
Region of study	
Australia	3
Europe/UK	4
US	2
Middle-East	1
Recruitment	
Hospitals/clinics	3
Private Practices/GPs	3
Pregnancy loss support group	2
Pregnancy loss clinic	1
Newspapers	1
Community locations (noticeboards, libraries, centres, pharmacies, shops)	2
Snowball sampling/word of mouth	5
Time since pregnancy loss	
0–8 weeks	4
8 weeks-6 months	6
6 months-1 year	5
1 year-2 years	3
2 years–3 years	2
> 3 years	1
Time of gestation at which loss occurred	
6–12 weeks	3
13–20 weeks	4
21 weeks–30 weeks	2
31 weeks–40 weeks	3
> 40 weeks	3

^a^ If numbers total more or less than 10 then the characteristics of a study were unknown or were relevant in two groups

^b^ The sample size for studies assessing couples is halved so that the results for men only are displayed

Sample sizes of male participants included in the studies ranged from four to 14, but were most commonly between 10 and 14. Time since the pregnancy loss occurred and when they participated in the research varied, but was most commonly between 8 weeks and 6 months. The time of gestation that the pregnancy loss occurred was most commonly between 13 and 20 weeks. Seven of the studies adopted a phenomenological approach using thematic analysis, in which the researchers undertook semi or unstructured interviews with each participant. Two of the studies had an interpretive narrative design, while one study was a self-reported questionnaire of open-ended questions.

### Outcomes of qualitative studies

Seven studies which aimed to describe men’s experiences of pregnancy loss either in the context of another pregnancy or as an occurrence in itself reported similar themes, particularly that of men feeling that their role was primarily as a ‘supporter’ to their female partner [10, 39–44]. These studies examined the normative social expectation that males ought to be caretakers and sources of strength, together with the associated outcome of feeling unable to express their emotions due to the expectation that they maintain control of the situation and be a comfort to their female partner. These studies found that while many men would make the effort to appear overtly ‘strong’ and return to their regular life as a coping strategy, they nevertheless experienced internal feelings of stress and vulnerability which intensified their role of ‘protector’ in subsequent pregnancies following a previous loss. Furthermore, men often reported feeling unable to respond to their female partners with emotional support, preferring instead to respond with instrumental support by absorbing practical strain such as paying the bills, in the hope of maintaining routine and ‘protecting’ their female partners.

Five qualitative studies also identified the lack of social recognition that many men felt in dealing with a pregnancy loss, describing feeling overlooked, alienated and marginalised in comparison to their female partners who had suffered the physical experience and whose pain was more visible [10, 39, 43–45]. Studies also focused on different expressions of male grief when compared to their female partners, in that while male participants expressed feelings that are typical of the grief and bereavement process such as sadness and uncertainty, they tended to report less intense feelings that lasted a shorter amount of time. Furthermore, the predominant emotions reported by men were those of frustration and helplessness. This finding reflected that of the quantitative studies, as discussed above.

Another theme identified from the qualitative studies was the idea of a loss of identity due to what the pregnancy had come to mean to men who had experienced pregnancy loss. The studies found that most men anticipated a healthy pregnancy, so participants in three studies reported a struggle in recognising their transition into fatherhood, and questioned if they had the right to the ‘father’ title following a loss [41, 43, 46]. Male participants described a deep sense of loss for the hopes and dreams they had visualised for their baby, and also the hopes they had invested in the prospect of being a parent, particularly after bonding with images of their baby through ultrasound, gaining a sense of their physical features, and becoming ‘mentally engaged’ with the idea of being a father. Participants in Murphy’s [45] study, for example, explained that having children was viewed as being ‘normal’ in society, so miscarriage therefore threatened the process of becoming a father and having a family.

A common coping mechanism that was also described in nine of the studies was that of active-avoidance, whereby men withdrew emotionally and/or physically by choosing to return to work early and immersing themselves in their work as a distraction. Khan et al. [47] touched on a health impact of pregnancy loss when they found that four of the nine men in their study tried to ignore their feelings by focusing on other distractions such as smoking. Participants in O’Leary and Thorwick’s [10] study also touched on physical health issues when they described feeling both physically and emotionally exhausted following a miscarriage, which they attempted to manage by keeping busy and going to work. In some studies, men stated that while work was a temporary distraction, the combination of both household and work pressures wore them down [10].

### Quality and bias

The overall quality of the studies was impacted by several issues relating to participant selection, and the final participant sample – issues which are likely to impact knowledge in relation to men’s health outcomes following pregnancy loss. In particular, almost all of the quantitative studies [7, 16–18, 23–35, 37, 38] and a smaller number of the qualitative studies [39, 40, 46] primarily included men as part of a heterosexual couple, with men often recruited through their female partners. Indeed, several studies specifically only included men in order to compare outcomes with their female partners [7, 23, 30, 40]. While these studies certainly contribute to literature in this area, they are limited in terms of what they can tell us specifically in relation to men’s individual responses, and offer little insight into the experiences of men who are single or gay (as discussed further below). Relatively few quantitative [18, 25, 27, 32, 33, 35] or qualitative studies [10, 42–45, 47] recruited only fathers, or aimed specifically to explore men’s experiences of loss. Many studies made no mention of culture or ethnicity at all [10, 16, 17, 32, 34, 36, 43–45], or included a majority of Caucasian participants or those who spoke English [28, 31]. This method of recruitment and subsequent sample may present issues for data collection concerning the retrospective health impacts of the loss. Similarly, the fact that the majority of the studies were conducted in middle to high income countries also presents a bias in the evidence base, given that 98% of all stillbirths occur in either low or middle income countries [3].

## Discussion

Overall, this review found several common themes across studies that have explored men’s experiences of pregnancy loss. Specifically, findings indicated that men typically feel as though they need to take on a ‘supporter’ role for their female partner, which may come at the expense of their own health and wellbeing [10, 39–44]. In addition, the existing body of research indicates that pregnancy loss may lead to a loss of identity related to both the anticipated father role, and the grief and loss associated with the changes which may come after a pregnancy loss [41, 43, 46]. Finally, existing research mirrors that with women in finding that pregnancy loss and associated grief lack social recognition, leading to disenfranchised grief for men, as well as challenges accessing support and often negative impacts upon relationships [10, 39, 43–45].

While the results of this review indicate that the feelings associated with pregnancy loss are often very similar between men and women, the manifestations of these feelings are typically different, indicating that men’s health and wellbeing is an important research area in itself. The lack of knowledge of these issues amongst some health providers and family and friends of couples who have experienced a loss can lead to helplessness, marginalisation and the feeling of being alone in their grief [10, 17, 24, 28, 39].

Many studies focussing on heterosexual cisgender men have tended to infer behaviour and emotions from the female partner’s reports, due in part to a socio-cultural belief that men may be less willing to communicate and that pregnancy loss has a greater impact on women [33]. There are also some methodological issues in previous research concerning men’s experience of pregnancy loss. For example, men have tended to be interviewed as part of a couple, and those studies which are solely from the man’s perspective tend to be in the context of a subsequent pregnancy and/or have some issues with generalisability due to population, sampling, and response bias. Moreover, most qualitative studies of men’s experiences have small sample sizes, are often case-studies, and have often used convenience/snowball sampling to obtain participants through means of recruitment via health centres or self-help groups. This may indicate that the men interviewed already have a vested interest in their health and wellbeing following the pregnancy loss, and may not be representative of the general population of men who are experiencing the impact of a pregnancy loss.

As discussed previously, a common theme evident in seven studies involving men and pregnancy loss is one of being a supporter and ‘remaining strong’ in the face of loss. Therefore, there may also be a social desirability bias inherent in some men’s responses, in that they may not fully disclose their feelings and the challenges they face as they do not want to appear weak or vulnerable. In terms of a cultural bias, while there are some studies from different countries and different cultures including the UK, the US, Japan, Australia, Israel and Portugal that explore pregnancy loss from a male perspective, they do not explore how reactions to a loss may be attributed to cultural differences (for example in understandings of what loss and grieving means, death rituals, and cultural constructions of relationship meaning within families and parents) [48].

Another gap in the literature pertains to gay and/or transgender men’s experiences of pregnancy loss. Exceptions to this include the work of Ziv and Freund-Eschar [49] who conducted in-depth interviews with eight gay couples from Tel-Aviv expecting a baby through surrogacy in either the United States or India. As part of their analysis, Ziv and Freund-Eschar touched on the impact of pregnancy loss in describing one participant’s frustration and anxiety with regard to the ways in which surrogacy clinic practices did not allow for emotions to be expressed in regards to a miscarriage, with the participant reporting disappointment over time wasted. Riggs, Due and Power [50] also conducted interviews with 12 gay men who had undertaken surrogacy arrangements in India. A key issue that arose in the interviews was the lack of sensitivity shown by clinics following a pregnancy loss. One participant described the initial response of the clinic as talking about finding another surrogate who had a high success rate, rather than acknowledging their grief and assisting in organising the funeral. Finally, in regards to transgender men, Ellis, Wojnar and Pettinato [51] reported on a qualitative study of eight trans or gender diverse men who had undertaken a pregnancy. Half of these men had experienced at least one miscarriage, with one participant reporting that they felt betrayed by their body as a result of a miscarriage. This small number of papers suggests the importance of future research that focuses specifically on the health and psychological impacts of pregnancy loss for gay and/or transgender men, however none met the inclusion criteria for this paper with respect to a specific focus on pregnancy loss.

There are no previous systematic reviews which aim to describe the nature and characteristics of pregnancy loss as experienced by men with a specific focus on health and wellbeing. The search strategy only included peer-reviewed studies that were written in English, so this may be considered as a source of bias and future studies may benefit from including grey literature as well as literature in languages other than English. The scope of the review was necessarily broad which reflects the paucity of the current literature base on the subject. Due to this fact, a quality screening was not undertaken and a meta-analysis was not done due to the heterogeneity among studies. Future studies may be able to utilise meta-analysis to examine psychological impacts in particular in more depth.

## Conclusions

Future empirical research would benefit from longitudinal studies with less of a focus on measurable psychological aspects of pregnancy loss on men and an increased focus on other aspects of health and wellbeing that may be affected by such a loss, for example physical health. Most studies that encompass different characteristics of health tend to be qualitative. The development or use of a psychometrically valid measure which incorporates non-psychological aspects of health and can be used to measure a larger sample would be beneficial in capturing information that may be more generalisable. It is important to acknowledge the impact that avoidance and coping behaviours may have on men and their partners as a result of a pregnancy loss. These behaviours may include focusing on work as a distraction, and increasing risk behaviours such as excessive alcohol consumption, smoking and drug. These avoidance and coping behaviours may exacerbate the experience of the loss and lead to relationship breakdowns and prolonged grief.

Studies that include a larger sample of men from varying cultural and religious backgrounds to show how the impact of a pregnancy loss may differ in relation to culture and context may also be beneficial. Culture can strongly influence and define grieving processes, and provides a framework within which human relationships vary and are given meaning [3]. The process of acculturation whereby people move to a different cultural setting may also influence the role of culture as findings may be similar to those who had always lived in that particular cultural setting. With this in mind, it is important to understand how men from varying cultures are impacted upon by grief following a pregnancy loss, and how they deal with this grief in their specific cultural contexts. It is also vital to explore pregnancy loss from the perspective of non-heterosexual non-cisgender men, as research with these populations is lacking. Approaching men’s experiences of pregnancy loss from a biopsychosocial perspective may lead to a better understanding in health care of how pregnancy loss may impact on men both physically and mentally, which may influence the development of improved practices and resources.

## Appendix

**Table 6:** Studies included from systematic searches

Author	Research Aims	Participants/Setting	Method/Design	Results/Conclusions
1. Quantitative studies				
Alderman et al. [23]	To study the psychological experience of a miscarriage and to determine if women and their partners experience the loss differently	19 Caucasian married couples (10 experienced a miscarriage in their first pregnancy), recruitment unknown but undertaken in the US	Questionnaires using psychological instruments: The Grief Experience Inventory-Loss Version and the Impact of Event Scale	Men and women’s overall response patterns were different. Men reported less grief and stress than their partners and men were less willing to admit their feelings. Men had elevated results on the Avoidance scale.
Armstrong [31]	To evaluate the association between previous perinatal loss and parents’ levels of depression, anxiety and prenatal attachment	103 couples in their second trimester (40 had a prior perinatal loss, 33 first time pregnancy, 30 with a history of successful pregnancies), prenatal clinics, education classes, private medical practices and internet message boards in the US	Structured questionnaires in person or over the telephone measured depressive symptoms (Center for Epidemiological Studies-Depression Scale), anxiety (Pregnancy Outcome Questionnaire) and prenatal attachment (Prenatal Attachment Inventory)	Couples with a history of perinatal loss had higher depressive symptoms and pregnancy-specific anxiety (Fathers reported less mothers). Perinatal attachment did not differ between groups (Fathers had lower levels of prenatal attachment than mothers).
Beutel et al. [24]	To ascertain similarities and differences in couples’ grief and depressive reactions following a miscarriage	56 couples from Germany (mean age of men was 33), experienced a spontaneous abortion between 6 and 16 weeks (*M* = 10), 48% had other children, 18% of women had previous miscarriages	Controlled follow-up study at 6 and 12 months after a miscarriage using standardised questionnaires measuring depression (von Zerssen Depression Scale), physical complaints (The Complaint List), anxiety (State-Trait Anxiety Inventory) and grief (Munich Grief Scale)	Men were found to grieve less intensely and less enduringly then women, the manner in which grief is experienced is similar however men cry less and feel less need to talk about it, men feel burdened by their partners grief, conflicting reactions affect couple interactions
Cumming et al. [7]	To examine the emotional burden of miscarriage for women and their partners, measuring anxiety and depression over 13 months	Complete data from 133 men and 273 women from three Scottish Early Pregnancy Assessment Units	Prospective study with follow up at 6 and 13 months after miscarriage, the hospital anxiety and depression scale (HADS) was the main outcome measure	Anxiety was a higher overall clinical burden than depression and men reported lower levels of anxiety and depression than women, a greater level of adjustment over time was reported by women
Daly et al. [36]	To determine the psychological morbidity among the male partners of women who had miscarried	25 men whose female partners had miscarried within the previous 6 weeks. Recruited from a miscarriage clinic in Dublin, Ireland where they were attending with their partners	Structured interviews including the Hospital Anxiety and Depression Scale which measures anxiety and depressive symptoms	50% of males had evidence of significant psychological morbidity following miscarriage, only 32% of men were able to find support for themselves
Franche & Mikail [30]	To compare emotional adjustment of men and women with and without pregnancy loss (in context of current pregnancy). Comparisons included between men and women in response to pregnancy loss.	28 men whose female partners have experienced pregnancy loss and were not pregnancy gain. Recruited from hospital and physicians in Canada.	Quantitative cross sectional design, using measures of depression (Beck Depression Inventory) and anxiety (State-Trait Anxiety Inventory).	Women scored higher on depression measures than did their male partners.
Johnson & Baker [33]	To examine if men’s coping response during pregnancy, childbirth and or miscarriage predict psychological outcomes at the time of childbirth/miscarriage or 1 year later and establish any changes in coping repertoire	332 expectant fathers (68 pregnancies ended due to miscarriage between 6 and 24 weeks gestation and 100 couples had suffered a miscarriage previously). Unclear of recruitment processes or setting	Longitudinal design: Measures of stress (Impact of Event Scale), anxiety (State-Trait Anxiety Inventory), depression (Beck Depression Inventory) and coping (Coping Response Inventory) during pregnancy, following childbirth or miscarriage and 1 year later	All psychological outcomes increased at childbirth/miscarriage compared with pregnancy, then decreased at 1 year. Approach-oriented strategies e.g. problem solving and support seeking are used less following a negative pregnancy outcome, higher avoidance coping following miscarriage
Kagami et al. [25]	To examine the effects of recurrent pregnancy loss on the psychological adjustment and psychosocial stress on couples	76 couples in Japan who visited the outpatient clinic of a tertiary hospital (Keio University Hospital)	Self-administered questionnaires assessing recurrent pregnancy loss associated stress, quality of the marital relationship (Quality Marital Index), depression (Beck Depression Index) and anxiety (State-Trait Anxiety Inventory)	Men showed significantly lower levels of depression, anxiety and stress compared with women, depressed and anxious women more likely to be unsatisfied with partner’s support, men reluctant to exhibit their negative feelings, men showed increased active-avoidance coping (e.g., returning to work)
Kong et al. [26]	To explore men’s psychological reaction following their female partner’s miscarriage and investigate similarities and differences	83 couples who had been admitted to a university-affiliated tertiary referral hospital in Hong Kong with a miscarriage over a 1 year recruitment period	Prospective 1 year longitudinal observational study: psychological reactions assessed immediately and at 3, 6 and 12 months after miscarriage using the 12 item General Health Questionnaire (GHQ-12) and the Beck Depression Inventory (BDI). Questionnaires were completed independently	A large amount of men scored high in the GHQ-12 and 16.9% scored high in the BDI immediately after miscarriage (associated with a planned pregnancy) but this strongly decreased in the first 3 months and then plateaued, men scored significantly lower than women 1 year after miscarriage, psychological impact was less enduring for men
Lin & Lasker [27]	To explore the patterns of grief reaction following a pregnancy loss to see if patterns were different than those commonly noted in the literature	138 women and 56 of their male partners in Pennsylvania, USA who had experienced a pregnancy loss, recruited from a non-hospital based midwifery centre, ob/gyn private practices, four hospital ob/gyn clinics, a city health bureau and a social service agency	Longitudinal: three waves of the Perinatal Grief Scale over the course of 2 years (2 months, 1 year and 2 years after the loss)	Large variety of grief patterns found, which were more complicated than had previously been described in the literature, men experienced lower levels of grief after pregnancy loss than women, women show greater distress than men shortly after the loss but there is no change in adjustment after 1 year between men and women
McGreal et al. [34]	To examine whether male and female partners had different coping behaviours following perinatal death	17 males and 35 females who had approached the Bonnie Babes Association in Australia for assistance in coping with the stress of pregnancy loss. Time since the pregnancy loss varied from less than 12 months to 5 years	Self-administered questionnaire about coping behaviours (The Coping Scale for Adults). This was completed individually and in confidence.	Results suggested gender differences in coping strategies; the highest coping strategies for men were work hard, problem solve, use friendships, indulge in wishful thinking, worry, focus on the positive, tension reduction and keep to oneself; the lowest were spiritual support, social action and physical recreation
Puddifoot & Johnson [35]	To measure characteristics of male response following their partner’s miscarriage	323 male partners of women who miscarried within 8 weeks of the study. Recruited from north-east England and the Midlands.	Self-administered Perinatal Grief Scale	Men scored similar to female cohorts on the grief scale, characteristic differences in the way grief was handled e.g. less immediate active grief, duration of the pregnancy and seeing the ultrasound increased levels of grief
Serrano & Lima [29]	To describe the consequences of recurrent pregnancy loss on couples’ relationships and explore gender differences in attitudes and grief intensity	30 couples with at least 3 recurrent miscarriages and no living children, time interval between pregnancy loss and data collection was at least 3 months, most had losses prior to 13 weeks gestation, 2 couples were Black, 28 couples were Caucasian and all were recruited at the Recurrent Miscarriage Clinic in Lisbon, Portugal.	Self-administered questionnaires assessing psychological and relational impact (Impact of Events Scale and Perinatal Grief Scale) and measuring the quality of the couple’s relationship (Intimate Relationship Scale and Partnership Questionnaire). Members of each couple answered the questionnaires separately	Men grieve less intensely, relationships were not usually adversely affected by miscarriage but couples described sexual changes with grief being related to the quality of their sex life for men and quality of communication for women
Turton et al. [32]	To assess the psychological morbidity of fathers in a pregnancy subsequent to a stillbirth, test within-couple effects and identify risk factors	38 pregnant couples whose previous pregnancy had ended in stillbirth and 38 pair matched controls, antenatal clinics in 3 general hospitals in the UK	Psychological assessments antenatally and at 6 weeks, 6 months and 1 year postnatally: antenatal questionnaire about medical history, socio-economic status and stillbirth; Beck Depression Inventory; Spielberger State-Trait Inventory (anxiety measure); PTSD-I Interview; and Golombok Rust Inventory of Marital Satisfaction	Fathers in the index group experienced significant levels of anxiety and PTSD antenatally but all symptoms subsided postnatally. Fathers experienced greater anxiety when a subsequent pregnancy following stillbirth was delayed. Fathers may be vulnerable to psychological distress during a pregnancy following a stillbirth
Vance et al. [38]	To examine patterns of anxiety, depression and alcohol use in couples following stillbirth, neonatal death or sudden infant death syndrome	138 bereaved and 156 non-bereaved couples. Bereaved couples were referred by seven obstetric hospitals in south-east Queensland, Australia and they were matched with non-bereaved couples recruited through the same hospitals	Prospective study: Couples completed standardised interviews at 2, 8, 15 and 30 months post-loss that measured self-reported distress (Delusions Symptoms State Inventory to measure anxiety and depression, questions about frequency and quantity of alcohol consumption to measure alcohol use and seven items from the satisfaction subscale of the Spanier Dyadic Adjustment Scale to measure marital satisfaction)	Both partners were rarely distressed in either group, father only distress ranged from 7% to 15% peaking at 30 months, distress more common feature in bereaved couples, fathers less likely to be distressed, 7%–12.3% of bereaved fathers met the criterion for heavy alcohol usage compared to 4.7%–5.8% for non-bereaved fathers
Zeanah et al. [28]	To investigate factors that may influence mothers’ and fathers’ adaptation following perinatal loss and the differences between them	82 mothers and 47 of their male partners who had experienced a perinatal loss 2 months previously and were between 20 and 44 weeks gestation, recruited from a single tertiary referral hospital in New England, USA.	Assessments conducted by researchers in the family’s home: Ego Strength Scale (parental personality), Nethelp, Dyadic Adjustment Scale, Life Experiences Survey (parental social characteristics), Beck Depression Inventory, Grief Experience Inventory-perinatal, Perinatal Grief Scale (grief and affective symptoms). Members of each couple answered the questionnaires separately	Fathers had lower levels of grief than mothers in 75% of the sample population; fathers with less ego strength, less social support and more stressful live events had higher levels of grief; personality characteristics were the strongest predictors of grief intensity
2. Qualitative studies				
Abboud & Liamputtong [39]	To examine the means by which women and their partners cope with a miscarriage	Six women and their partners from ethnic backgrounds (Middle-East and Philippines) living in Melbourne, Australia. All Christian, recruited via snowballing and one couple from a GP referral	Phenomenological approach using thematic analysis: 40–90 min in-depth interviews in participant’s homes, both members of each couple interviewed separately	All men mentioned their role was to support, coped by trying not to make it a big issue and returning to ‘normal’, men described family members as assisting partners but not themselves, most men did not believe talking to others would help so they did not
Abboud & Liamputtong [40]	To examine the experiences and perceptions of women and their partners who have suffered a miscarriage	Six women and their partners from ethnic backgrounds (Middle-East and Philippines) living in Melbourne, Australia. All Christian, recruited via snowballing and one couple from a GP referral	Phenomenological approach using thematic analysis: Unstructured in-depth interviews in participant’s homes, both members of each couple interviewed separately	Men experienced less intense feelings for a shorter period of time than women, men stated that their role was to support and encourage and they had to consider their partner first, most men stated they were happy and no longer though about the miscarriage
Armstrong [41]	To explore fathers’ experiences of pregnancy after a prior perinatal loss	Four men whose wives were currently pregnant following a previous loss in the second or third trimester, recruited through healthcare providers at medical practices in two US cities	Phenomenological: 45–90 min unstructured and semi-structured in-depth interviews (initial interview about the loss and experience of current pregnancy and second interview 3–4 weeks later for ongoing analysis	All fathers expressed anxiety and a heightened sense of risk about the outcome of the subsequent pregnancy. Themes included: intensity of the experience, dealing with grief, supporting their partner, replacement of the loss and importance of milestones
Bonnette & Broom [42]	To explore men’s experiences of stillbirth and how they experienced fathering and grief	12 men who were recruited over a 6 month period by purposive and snowball sampling via posters on community noticeboards, libraries, community centres, pharmacies and shops throughout regional New South Wales, Australia.	Qualitative in-depth 45 min to 2.5 h interviews	Identify as fathers in complex ways, expressing grief in the context of the ‘male role’ is problematic, fathering and grief are situated in a gendered dynamic
Hamama-Raz [46]	To examine the meaning of abortion amongst religious Jewish couples and how this meaning is expressed	Five couples: 3 Haredi (ultraorthodox) and 2 Dati-Leumi (national-religious representing the Zionist movement), had experienced a spontaneous abortion between the 10th and 18th week of pregnancy after a previous successful pregnancy, recruited in Israel via snowballing	Interpretive narrative study: 2 h semi-structured interviews by a female Hebrew social worker in their homes, both members of each couple interviewed separately	Both experienced spontaneous abortion as some kind of loss but expressed it differently (men saw is as a loss of potential), themes emerged: meaning of relationship with the foetus; doubts about parenthood; and crisis in faith.
Khan et al. [47]	To assess the emotional response of males whose female partners had suffered early pregnancy loss and establish if sufficient support services are provided (provide recommendations if necessary)	Nine Caucasian men attending a specialised Early Pregnancy Loss Clinic with their partners at Rotunda Hospital in Dublin, Ireland. Pregnancy loss occurred before 20 weeks gestation and the questionnaires were completed while in the waiting room 6–8 weeks following the pregnancy loss	Close and open-ended questionnaire	Men expressed feelings typical of grief and bereavement process such as sadness and uncertainty. Acceptance and depression were reported later. Pregnancy loss may represent a failure for men
McCreight [43]	To describe the experiences of men whose partner had experienced pregnancy loss	14 men who attended pregnancy loss self-help groups in Northern Ireland (range of gestational stage when pregnancy loss occurred was 7 weeks to 40 weeks and period since the loss ranged from 3 months to 20 years). Also 32 midwives and nurses to examine attitudes towards bereaved fathers	Observation and in-depth semi-structured interviews of a narrative nature (observation took place once a month over 3 years and interviews with 14 volunteers took place over those 3 years also). Most interviews took place in their homes.	Themes uncovered: self-blame, loss of identity, need to be appear strong, grief and anger. The perception of men as having the supportive role is unjustified, lack of legal recognition and institutional validation posed problems for their identity
Murphy [45]	To describe the experience of early miscarriage from a male perspective	Five men whose partners miscarried early more than 2 years prior to the interview. Snowballing used to find participants located in the UK.	Phenomenological approach: 30–60 min unstructured interviews	Themes emerged: feelings, loss, differences between men and women, staff action and attitudes, what to do, coping and time. Predominant feelings were frustration, helplessness and loneliness. Avoidance/ignoring was a common coping strategy
O’Leary & Thorwick [10]	To present information about fathers’ perspectives during the experience of a pregnancy following a prior perinatal loss	10 fathers who had experienced a loss within the prior year and were with the same partner in a subsequent pregnancy, obtained through friends of parents who had been involved in a pregnancy loss support group; notice in a bereavement newsletter; and staff at a perinatal centre	Descriptive phenomenology: 60–90 min one-on-one interviews in venues chosen by the fathers (i.e. home or clinic)	Four themes emerged: recognition-fathers need to be recognized by others, pre-occupation-conduct of their daily lives in disrupted, stoicism-unable to share fear and anxiety as they want to protect their partners, and support-societal pressure to be strong.
Samuelsson et al. [44]	To describe how fathers experienced losing a child as a result of intrauterine death	11 men whose offspring died between weeks 29 and 42 of pregnancy. Recruited in Sweden and interviewed 5 to 27 months after the loss	Phenomenological approach: 25 min to 2 h interviews mostly in their homes and some in a hospital	Strong feelings of frustration and helplessness, found meaning and relief in supporting their partner, the most important comfort was a good relationship with their partner, important to be able to grieve in their own way
3. Mixed methods studies				
Conway & Russell [16]	To investigate the grief responses of women and their partners following a miscarriage and discover if support received met their needs	39 women and 32 of their male partners who had experienced a pregnancy loss between 5 and 16 weeks gestation. Recruitment was from four major obstetrics hospitals and one district hospital in Sydney, Australia (an accurate participation rate was only obtained from one hospital), 11 GPs also approached patients after a miscarriage	Prospective study: Self-administered initial questionnaire including the Perinatal Grief Scale which was completed within 3 weeks of the miscarriage to minimise retrospectivity (80% of the questions were closed questions), follow-up self-administered questionnaire including the Perinatal Grief Scale 2–4 months after the miscarriage (48% of the questions were open-ended)	On the initial questionnaire, men scored significantly higher on the Perinatal Grief Scale than women, they were also higher on the follow-up questionnaire but not significantly, high attrition rate between initial and follow-up, the greatest difference was on the active-grief subscale (men tend to suppress outward signs of grief)
DeFrain et al. [17]	To examine the emotional, social and physical effects of miscarriage on family members and suggest implications for health professionals	21 fathers and 272 mothers who had experienced a miscarriage between 1 month and 42 years prior to completing the study (*M* = 5.4 years), recruited through 30 newspapers in the U.S. (people in 32 states participated)	A 23 page questionnaire was developed after 20 pilot interviews were conducted (63% of the questions were quantitative, 37% were qualitative)	Many fathers and mothers reported flashbacks and nightmares, they turned to each other for support, themes included: guilt and blame; reactions to crisis and pain; perceptions of others minimizing the miscarriage; and returning to normal
Johnson & Puddifoot [18]	To examine the psychological impact on the male partners of women who have miscarried	126 men in Britain whose partners had suffered a spontaneous abortion prior to the start of the 25th week of pregnancy. Recruited via referrals from gynaecological wards of general hospitals in the north-east of England	Opportunity sample survey supplemented by 10 semi-structured interviews with volunteers from the sample (42 volunteered). Two standard scales were used: the Perinatal Grief Scale and the Impact of Events Scale. Participants completed the questionnaires in their own time	High levels of grief were found that were felt strongly in the short term which affected their daily lives and relationship, the duration of the pregnancy and experience of ultrasound images were associated with higher levels of grief and stress, scores on the avoidance subscale were considerably high.

## Abbreviations

EU: European Union
JBI: Joanna Briggs Institute
PRISMA: Preferred Reporting Items for Systematic Reviews and Meta-Analyses
SCIE: Social Care Institute for Excellence
UK: United Kingdom
US: United States

## References

[1] Rinehart MS, Kiselica MS (2010). Helping men with the trauma of miscarriage. Psychother Theor Res.

[2] Brier N (2008). Grief following miscarriage: a comprehensive review of the literature. J Women’s Health.

[3] Blencowe H, Cousens S, Jassir F, Say L, Chou D, Mathers C, Hogan D, Shiekh S, Qureshi Z, You D, Lawn J (2016). National, regional and worldwide estimates of stillbirth rates in 2015, with trends from 2000: a systematic analysis. Lancet.

[4] Australian Institute of Health and Welfare (2014). Stillbirths in Australia1991–2009.Perinatal statistics series no. 29.

[5] Goksedef BPC, Kef S, Akca A, Bayik RNE, Cetin A (2011). Risk factors for rupture in tubal ectopic pregnancy: definition of the clinical findings. Eur J Obstet Gyn R B.

[6] Collins C, Riggs DW, Due C (2014). The impact of pregnancy loss on women’s adult relationships. Grief Matters.

[7] Cumming G, Klein S, Bolsover D, Lee A, Alexander D, Maclean M, Jurgens J (2007). The emotional burden of miscarriage for women and their partners: trajectories of anxiety and depression over 13 months. BJOG Int J Obstet Gynaecol.

[8] Harvey J, Creedy D, Moyle W (2001). Women’s experience of early miscarriage: a phenomenological study. Aust J Adv Nurs.

[9] Ujda RM, Bendiksen R (2000). Health care provider support and grief after perinatal loss: a qualitative study. Illness Crisis Loss.

[10] O'Leary J, Thorwick C (2006). Fathers’ perspectives during pregnancy, postperinatal loss. JOGNN.

[11] Moher D, Liberati A, Tetzlaff J, Altman DG (2009). Preferred reporting items for systematic reviews and meta-analyses: the PRISMA statement. Ann Intern Med.

[12] Joanna Briggs Institute (2014). Joanna Briggs Institute reviewers’ manual: 2014 edition.

[13] Rutter D, Francis J, Coren E, Fisher M (2010). SCIE systematic research reviews: guidelines.

[14] Broen AN, Moum T, Bödtker AS, Ekeberg Ö (2004). Psychological impact on women of miscarriage versus induced abortion: a 2-year follow-up study. Psychosom Med.

[15] Korenromp MJ, Page-Christiaens GC, van den Bout J, Mulder EJ, Hunfeld JA, Bilardo CM, Visser GH (2005). Psychological consequences of termination of pregnancy for fetal anomaly: similarities and differences between partners. Prenat Diagn.

[16] Conway K, Russell G (2000). Couples’ grief and experience of support in the aftermath of miscarriage. Br J Med Psychol.

[17] DeFrain J, Millspaugh E, Xie X (1996). The psychosocial effects of miscarriage: implications for health professionals. Fam Syst Health.

[18] Johnson MP, Puddifoot JE (1996). The grief response in the partners of women who miscarry. Br J Med Psychol.

[19] Beck AT, Ward C, Mendelson M (1961). Beck depression inventory (BDI). Arch Gen Psychiatry.

[20] Spielberger CD, Sydeman SJ, Maruish ME (1994). State-trait anxiety inventory and state-trait anger expression inventory. The use of psychological testing for treatment, planning and outcome assessment.

[21] Toedter LJ, Lasker JN, Alhadeff JM (1988). The Perinatal Grief Scale: development and initial validation. Am J Orthop.

[22] Horowitz M, Wilner N, Alvarez W (1979). Impact of event scale: a measure of subjective stress. Psychosom Med.

[23] Alderman L, Chisholm J, Denmark F, Salbod S (1998). Bereavement and stress of a miscarriage: as it affects the couple. OMEGA J Death Dying.

[24] Beutel M, Willner H, Deckardt R, Von Rad M, Weiner H (1996). Similarities and differences in couples’ grief reactions following a miscarriage: results from a longitudinal study. J Psychosom Res.

[25] Kagami M, Maruyama T, Koizumi T, Miyazaki K, Nishikawa-Uchida S, Oda H, Schmidt L (2012). Psychological adjustment and psychosocial stress among Japanese couples with a history of recurrent pregnancy loss. Hum Reprod.

[26] Kong G, Chung T, Lai B, Lok I (2010). Gender comparison of psychological reaction after miscarriage: a 1-year longitudinal study. BJOG Int J Obstet Gynaecol.

[27] Lin SX, Lasker JN (1996). Patterns of grief reaction after pregnancy loss. Am J Orthop.

[28] Zeanah CH, Danis B, Hirshberg L, Dietz L (1995). Initial adaptation in mothers and fathers following perinatal loss. Infant Mental Health J.

[29] Serrano F, Lima ML (2006). Recurrent miscarriage: psychological and relational consequences for couples. Psychother Theor Res.

[30] Franche R, Mikail S (1999). The impact of perinatal loss on adjustment to subsequent pregnancy. Soc Sci Med.

[31] Armstrong DS (2002). Emotional distress and prenatal attachment in pregnancy after perinatal loss. J Nurs Scholarsh.

[32] Turton P, Badenhorst W, Hughes P, Ward J, Riches S, White S (2006). Psychological impact of stillbirth on fathers in the subsequent pregnancy and puerperium. Br J Psychiatry.

[33] Johnson MP, Baker SR (2004). Implications of coping repertoire as predictors of men's stress, anxiety and depression following pregnancy, childbirth and miscarriage: a longitudinal study. J Psychosom Obstet Gynaecol.

[34] McGreal D, Evans BJ, Burrows GD (1997). Gender differences in coping following loss of a child through miscarriage or stillbirth: a pilot study. Stress Med.

[35] Puddifoot JE, Johnson MP (1999). Active grief, despair and difficulty coping: some measured characteristics of male response following their partner's miscarriage. J Reprod Infant Psychol.

[36] Daly S, Harte L, O'beirne E, McGee H, Turner M (1996). Does miscarriage affect the father?. J Obstet Gynecol.

[37] Potvin L, Lasker J, Toedter L (1989). Measuring grief: a short version of the Perinatal Grief Scale. J Psychopathol Behav Assess.

[38] Vance JC, Boyle FM, Najman JM, Thearle MJ (2002). Couple distress after sudden infant or perinatal death: a 30-month follow up. J Paediatr Child Health.

[39] Abboud L, Liamputtong P (2005). When pregnancy fails: coping strategies, support networks and experiences with health care of ethnic women and their partners. J Reprod Infant Psychol.

[40] Abboud L, Liamputtong P (2002). Pregnancy loss. Soc Work Health Care.

[41] Armstrong D (2001). Exploring fathers’ experiences of pregnancy after a prior perinatal loss. MCN Am J Matern Child.

[42] Bonnette S, Broom A (2012). On grief, fathering and the male role in men's accounts of stillbirth. J Sociol.

[43] McCreight BS (2004). A grief ignored: narratives of pregnancy loss from a male perspective. Sociol Health Illn.

[44] Samuelsson M, Radestad I, Segesten K (2001). A waste of life: fathers’ experience of losing a child before birth. Birth Iss Perinat C.

[45] Murphy FA (1998). The experience of early miscarriage from a male perspective. J Clin Nurs.

[46] Hamama-Raz Y, Hemmendinger S, Buchbinder E (2010). The unifying difference: dyadic coping with spontaneous abortion among religious Jewish couples. Qual Health Res.

[47] Khan R, Drudy L, Sheehan J, Harrison R, Geary M (2004). Early pregnancy loss: how do men feel?. Ir Med J.

[48] Rosenblatt PC, Stroebe M, Hansson RO, Schut H, Stroebe W (2008). Grief across cultures: a review and research agenda. Handbook of bereavement research and practice: advances in theory and intervention.

[49] Ziv I, Freund-Eschar Y (2015). The pregnancy experience of gay couples expecting a child through overseas surrogacy. Fam J.

[50] Riggs DW, Due C, Power J (2015). Gay men's experiences of surrogacy clinics in India. J Fam Plann Reprod Health.

[51] Ellis SA, Wojnar DM, Pettinato M (2015). Conception, pregnancy, and birth experiences of male and gender variant gestational parents: it's how we could have a family. J Midwifery Womens Health.

